# Desmoplastic small round cell tumor is dependent on the EWS-WT1 transcription factor

**DOI:** 10.1038/s41389-020-0224-1

**Published:** 2020-04-28

**Authors:** Jenna M. Gedminas, Maggie H. Chasse, Mitchell McBrairty, Ian Beddows, Susan M. Kitchen-Goosen, Patrick J. Grohar

**Affiliations:** 1 Van Andel Research Institute, Grand Rapids, MI USA; 2 Division of Pediatric Hematology/Oncology, Helen DeVos Children’s Hospital, Grand Rapids, MI USA; 3 Division of Oncology, Children’s Hospital of Philadelphia, Philadelphia, PA USA

**Keywords:** Cancer, Cancer genomics, Paediatric cancer

## Abstract

Desmoplastic small round cell tumor (DSRCT) is a rare and aggressive soft-tissue malignancy with a poor overall survival and no effective therapeutic options. The tumor is believed to be dependent on the continued activity of the oncogenic EWS-WT1 transcription factor. However, the dependence of the tumor on EWS-WT1 has not been well established. In addition, there are no studies exploring the downstream transcriptional program across multiple cell lines. In this study, we have developed a novel approach to selectively silence EWS-WT1 without impacting either wild-type EWSR1 or WT1. We show a clear dependence of the tumor on EWS-WT1 in two different cell lines, BER and JN-DSCRT-1. In addition, we identify and validate important downstream target pathways commonly dysregulated in other translocation-positive sarcomas, including PRC2, mTOR, and TGFB. Surprisingly, there is striking overlap between the EWS-WT1 and EWS-FLI1 gene signatures, despite the fact that the DNA-binding domain of the fusion proteins, WT1 and FLI1, is structurally unique and classified as different types of transcription factors. This study provides important insight into the biology of this disease relative to other translocation-positive sarcomas, and the basis for the therapeutic targeting of EWS-WT1 for this disease that has limited therapeutic options.

## Introduction

Desmoplastic small round cell tumor (DSRCT) is a rare and aggressive soft-tissue malignancy. It typically occurs in adolescent and young adult males with a male-to-female ratio of 4:1^1^. DSRCT commonly presents with widely metastatic disease throughout the abdomen, as well as diffuse peritoneal carcinomatosis^2^. There is currently no standard recommendation for management, and despite aggressive multi-agent chemotherapy, and surgical resection, the median survival is 17 months with a 5-year survival rate of less than 20%^1^.

DSRCT is defined by the t(11;22)(p13;q12) translocation that fuses the N-terminal domain of EWSR1 to the C-terminal DNA-binding domain of WT1, creating the aberrant transcription factor, EWS-WT1, which is diagnostic for the disease^3^. Apart from this translocation, the disease is believed to have a low somatic mutation frequency, a finding similar to other translocation-positive pediatric sarcomas^4^. As evidence of this, a screen of 24 DSRCT samples using a panel of 29 genes did not identify any recurrent mutations^4^. Previous single-patient case reports have described amplification of *AURKB* and *MCL1*, as well as variants of unknown significance in *ARID1A* and *RUNX1*, but there have been no consistent recurrent mutations observed^5,6^.

The EWS-WT1 breakpoint can occur in introns 7–10 of *EWSR1* and invariably between exons 7 and 8 of *WT1*^2^. This fuses the potent transcriptional activation domain of EWSR1 with the DNA- binding domain of WT1^2^. It is believed that DSRCT cells depend on the continued activity of EWS-WT1, but there are no studies clearly establishing a dependence of the tumor on this fusion protein. Several studies have published individual downstream-induced targets of EWS-WT1, including PDGFA, IL2RB, and BAIAP3 based on inducible expression in various cell lines^7–9^. However, none of these studies led to cellular transformation following EWS-WT1 expression or established a comprehensive list of downstream EWS-WT1 targets in multiple patient-derived cell lines^3^.

In this study, we demonstrate the dependence of DSRCT on EWS-WT1 for cell survival. We identify a comprehensive list of downstream targets of the fusion protein in BER and JN-DSRCT-1 cells, and features of these two DSRCT cell lines that reflect the phenotype of the tumor^10^. Surprisingly, we identify a number of common features with other oncogenic fusion proteins, and EWS-FLI1 in particular, despite differences in the DNA-binding domain of the two proteins. Together, this study provides insight into the molecular pathogenesis of DSRCT and establishes EWS-WT1 as a bona fide drug target for this disease.

## Results and discussion

### DSRCT is dependent on the activity of EWS-WT1 for continued proliferation

**Fig. 1:**
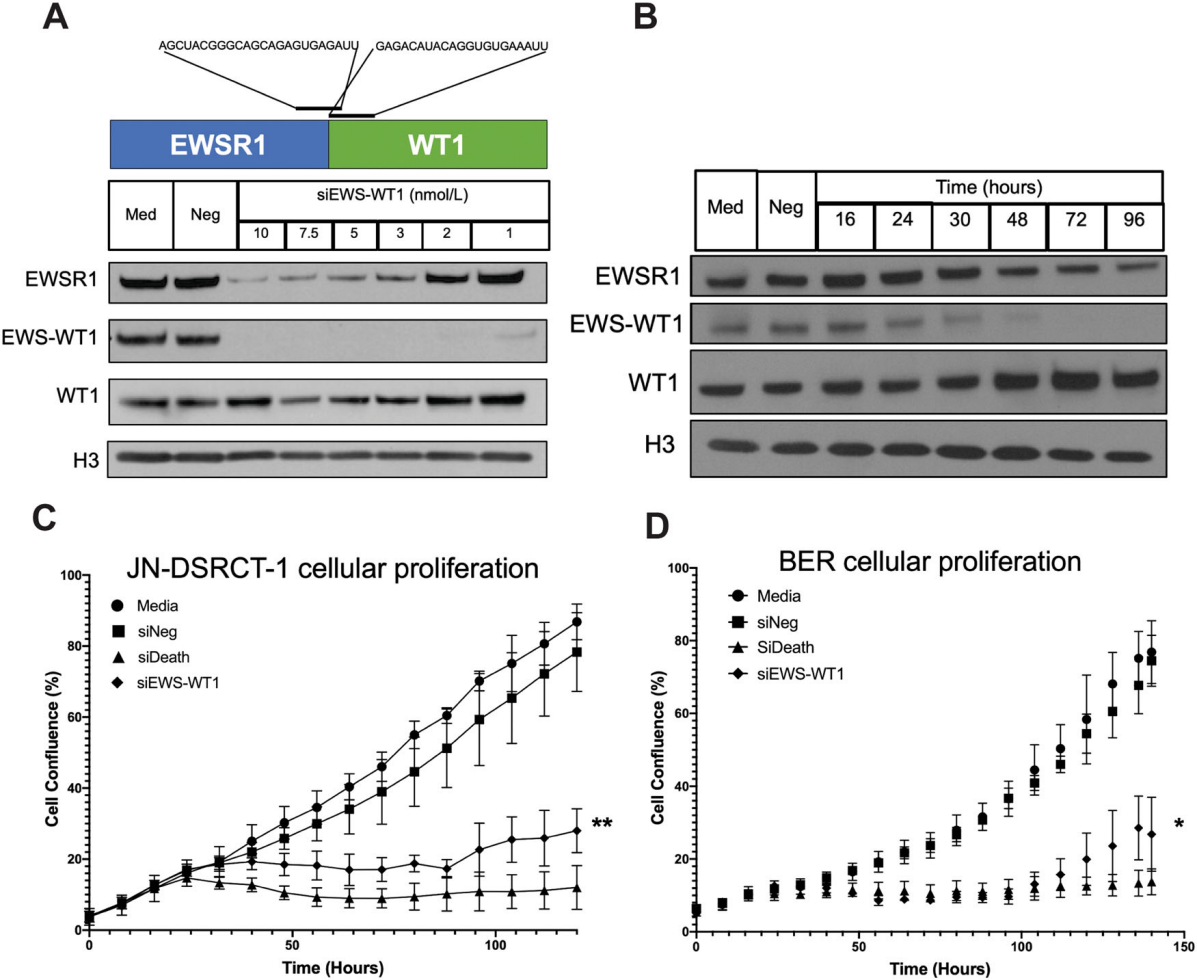
EWS-WT1 silencing leads to loss of DSRCT cell proliferation. **a** Western blot showing selective suppression of EWS-WT1 expression, but not EWSR1 or WT1 at 2 nmol/L compared with untreated cells (med.), a non-targeting siRNA (siNeg), or higher concentrations of siRNA that reduce the expression of wild-type EWSR1, wild-type WT1, and EWS-WT1. **b** Western blot demonstrates suppression of EWS-WT1 over time (hours) following siRNA silencing with 2 nmol/L siRNA in JN-DSRCT-1 relative to untreated cells (Med) or a non-targeting siRNA (Neg). Custom siRNA (Dharmacon) targeted to flank the EWS-WT1 breakpoint was complexed with RNAiMax (ThermoFisher) at the indicated concentration for 30 min at room temperature. Cells were added and incubated at 37 °C. The cells were collected, washed in PBS, and lysed in 4% lithium dodecyl sulfate (LDS) buffer. The protein was quantitated by bicinchoninic acid (BCA) assay (Pierce, Thermo-Scientific) and 30 µg were resolved on a NuPage 4–12% Bis–Tris gradient gel (Invitrogen) in 1× NuPage MOPS SDS Running Buffer (Invitrogen). The protein was transferred overnight to a nitrocellulose membrane at 20 V in 1× Tris-Glycine-SDS Buffer (Bio-Rad) containing 20% methanol. The membrane was blocked in 5% milk in TBS-T, and probed with EWSR1 (11910, Cell Signaling) and H3 (2650, Cell Signaling), and WT1 (sc-7385, Santa Cruz Biotechnology) antibodies. The results shown are representative of three independent experiments. **c**, **d** Silencing of EWS-WT1 (siEWS-WT1) leads to a loss of proliferation as measured by percent confluence relative to the positive control (siDeath), a non-targeting siRNA (siNeg), or untreated cells (Media) in JN-DSRCT-1 (**c**) and BER (**d**) cells. JN-DSRCT-1 or BER cells were incubated with complexed siRNA/lipid at 37 °C in the Incucyte Zoom^TM^ (Essen Bioscience) to measure confluence every 2 h. The results are displayed as the mean value with error bars representing standard deviation. Three individual replicates were plated in triplicate. *t* Tests were used to evaluate statistical significance from control, and adjustments were made to account for multiple comparisons. JN-DSRCT-1 cells were obtained from H. Li at New York University, and BER cells from the Christus Stehlin Foundation for Cancer Research and were confirmed mycoplasma negative. The presence of EWS-WT1 was confirmed by FISH (Supplementary Fig. 1). The cells were cultured at 37 °C, 5% CO_2_ in RPMI-1640 with 10% fetal bovine serum, 2 mM l-glutamine, 100 U/mL penicillin, and 100 μg/mL streptomycin.

In order to establish the dependence of DSRCT cells on EWS-WT1, we selectively silenced EWS-WT1 using siRNA. The challenge is that both wild-type EWSR1 and WT1 are expressed in the cell. To overcome this, we used two siRNA sequences (one on each side of the EWS-WT1 breakpoint) and titrated the concentration of the pooled siRNA to selectively suppress EWS-WT1 expression in BER and JN-DSRCT-1 without impacting wild-type EWSR1 or WT1 expression (Fig. 1a). Cell-line identity and the presence of the translocation was confirmed by FISH (Supplementary Fig. 1). EWS-WT1 expression was lost by 48 h, which correlated with a loss of proliferation in both cell lines (Fig. 1b–d). These data show that EWS-WT1 is both necessary and sufficient to drive DSRCT cell proliferation as knockdown of this fusion suppresses proliferation.

### Loss of EWS-WT1 activity leads to morphologic changes and growth arrest

**Fig. 2:**
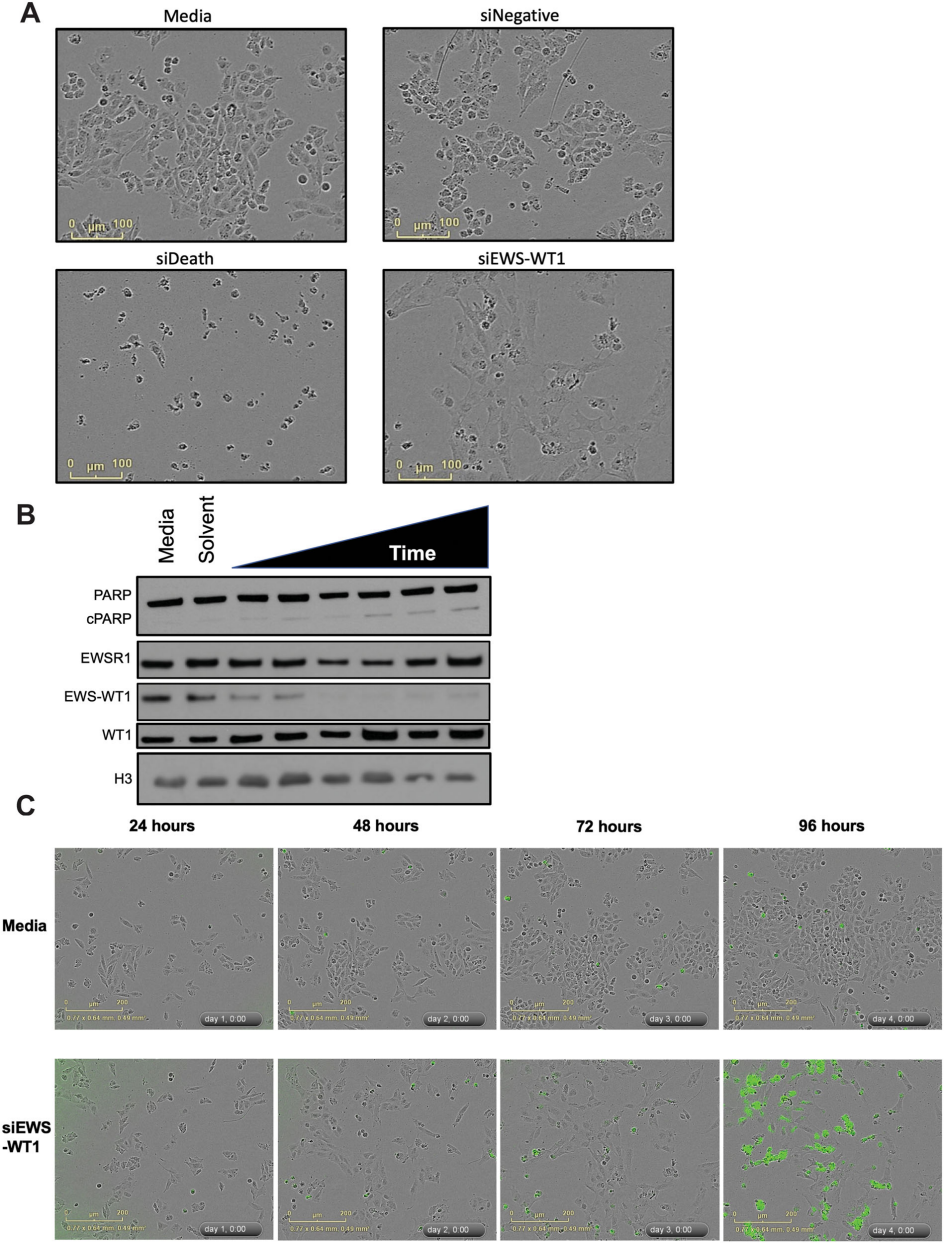
Loss of EWS-WT1 leads to morphologic changes and growth arrest in DSRCT cells. **a** JN-DSCRT-1 cells undergo a morphologic change with EWS-WT1 silencing (siEWS-WT1) compared with untreated cells (Medium), a non-targeting siRNA (siNeg), or a positive control siRNA (siDeath). **b**, **c** Western blot demonstrates accumulation of the apoptotic marker cleaved PARP following EWS-WT1 silencing (**b**) that parallels cleaved caspase 3/7 (green cells) after EWS-WT1 silencing in live cells (**c**) occurring long after the morphologic change. Lysates were collected at 16, 24, 30, 48, 72, and 96 h of exposure. Western blot probed with EWSR1 (11910, Cell Signaling), H3 (2650, Cell Signaling), and cPARP (9546, Cell Signaling) and WT1 (sc-7385, Santa Cruz Biotechnology) antibodies. Apoptosis was quantitated by measuring activation of CellEvent caspase 3/7 green detection reagent (ThermoFisher Scientific) in 10,000 cells/well in a 96-well plate following siRNA silencing of EWS-WT1.

We next explored the consequence of EWS-WT1 knockdown on the cellular phenotype. Forty-eight hours of siRNA silencing of EWS-WT1 led to growth arrest and a striking morphological change with the cells becoming enlarged and flattened relative to control (Fig. 2a). With continued suppression, there was clear induction of apoptosis as measured by the cleavage of PARP by western blot analysis by 60–72 h (Fig. 2b). This induction of apoptosis was confirmed using a fluorescent marker of cleaved caspase 3/7, which showed an increase in focal fluorescence following loss of EWS-WT1 compared with control cells (Fig. 2c). Importantly, apoptosis occurred long after growth arrest, indicating that this is likely a secondary effect rather than a direct effect of EWS-WT1 loss.

### EWS-WT1 loss alters the transcriptome of DSRCT cells

**Fig. 3:**
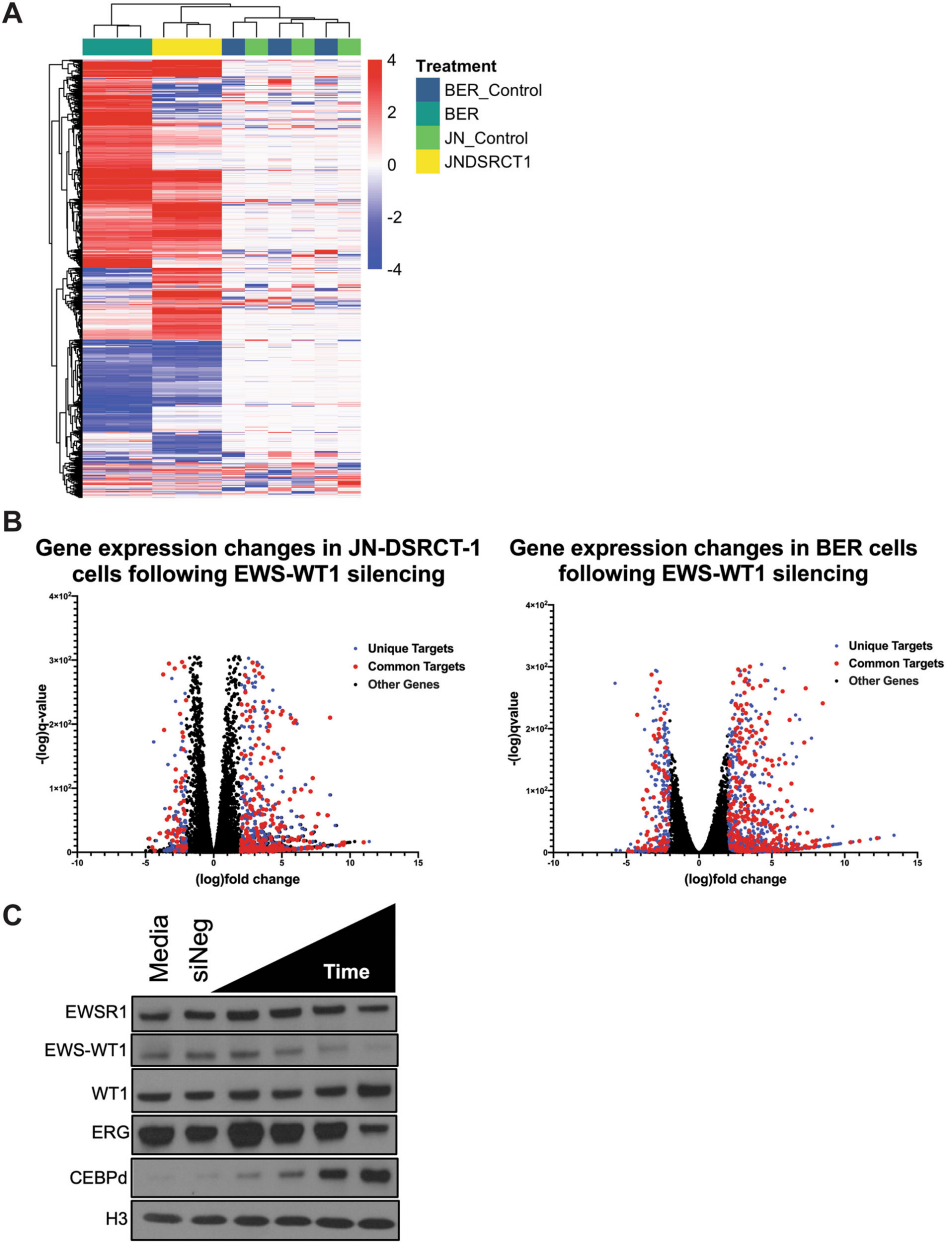
EWS-WT1 loss leads to large-scale gene expression changes in DSRCT cells. **a** Hierarchical clustering of gene expression changes following silencing of EWS-WT1 in JN-DSRCT-1 cells (JNDSRCT1) or BER cells (BER) relative to a non-targeting control siRNA in the same cells (JN_control or BER_control). Data represent 1879 gene expression changes that are either increased (red) or decreased (blue) by a log FC of >2. **b** Volcano plots showing the magnitude of gene expression change as a function of *q* value (false discovery rate-adjusted *P* value) with silencing. Gene expression changes are both common (red) across the two cell lines and unique to the cell line (blue). More genes are repressed by EWS-WT1 than are induced. Libraries were prepared from 500 ng of total RNA using the KAPA-stranded mRNAseq Kit (v5.17). RNA was sheared to 300–400 bp. Prior to PCR amplification, cDNA fragments were ligated to Bio Scientific NEXTflex dual adapters. Quality and quantity were determined using a combination of Agilent DNA High Sensitivity chip, QuantiFluor^®^ dsDNA System, and Kapa Illumina Library Quantification qPCR assays. Individually indexed libraries were pooled, and 75-bp, paired-end sequencing was performed on an Illumina NextSeq 500 sequencer using a 75-bp HO sequencing kit (v2). Base calling was done by Illumina NextSeq Control Software (NCS) v2.0 and demultiplexed to FastQ format with bcl2fastq v1.9.0 (Illumina Inc.). Reads were aligned to hg38 using STAR (v2.5.2b) with options –twopassMode Basic –quantMode GeneCounts. The data were filtered for a minimum of ten counts per million in at least one sample. Differential expression analysis was carried out using Deseq2 (v1.24.0) with apeglm (v1.6.0) applied to account for absolute magnitude of gene expression. Significant genes were determined using a cutoff of *q* value < 0.05^11^. Heatmaps were generated using pheatmap package (v1.0.12) in R (v3.6.1). Analysis was performed on three biological replicates of each sample. All samples were collected at the same time, and RNA was isolated on the QiaCube (Qiagen) in batches after sample randomization to avoid batch effect. The defined EWS-WT1 gene signature consists of genes with a log-fold change of 2 or greater in both cell lines following silencing. **c** Western blot of EWS-WT1 silencing confirms repression (ERG) and induction (CEBPD) of targets. Lysates collected at 16, 24, 30, and 48 h of exposure. Western blot probed with EWSR1 (11910, Cell Signaling) and H3 (2650, Cell Signaling), WT1 (sc-7385 Santa Cruz Biotechnology), ERG (ab92513, Abcam), and CEBPD (ab65081, Abcam) antibodies.

Although a number of individual targets of EWS-WT1 have been reported in the literature, the gene signature of the fusion protein and a comprehensive list of downstream targets has not been established in more than one cell line^5,6^. RNA sequencing was performed after 48 h of siRNA silencing of EWS-WT1 to ensure complete silencing while minimizing secondary effects on gene expression that would occur with prolonged silencing (Fig. 1b). We identified a total of 1879 genes across both cell lines that changed in expression by >2 log-fold change (FC) (Fig. 3a). Importantly, there were more large-magnitude gene expression changes that were unique to a given cell line than common in both models (Supplementary Tables 1–3). Furthermore, consistent with observations in other sarcomas, more genes were repressed than induced by EWS-WT1 (Fig. 3b)^12^. We defined a core gene signature consisting of 68 genes induced and 223 genes repressed by EWS-WT1 in both cell lines (Supplementary Table 4). Importantly, we found a number of targets previously identified in the literature, including FGFR4, JAK3, IGF signaling, and members of the Wnt pathway that showed a >2 log FC in both cell lines. Other previously identified targets of the fusion protein, such as PDGFA and BAIAP3, demonstrated >1.5 log FC in both cell lines^9,13–15^. Finally, we validated suppression of the protein expression of both an induced (ERG) and a repressed (CEBPD) target (Fig. 3c). This is the first comprehensive analysis of the EWS-WT1 gene signature in two different DSRCT cell lines.

### Functional GSEA suggests important features of the tumor

**Fig. 4:**
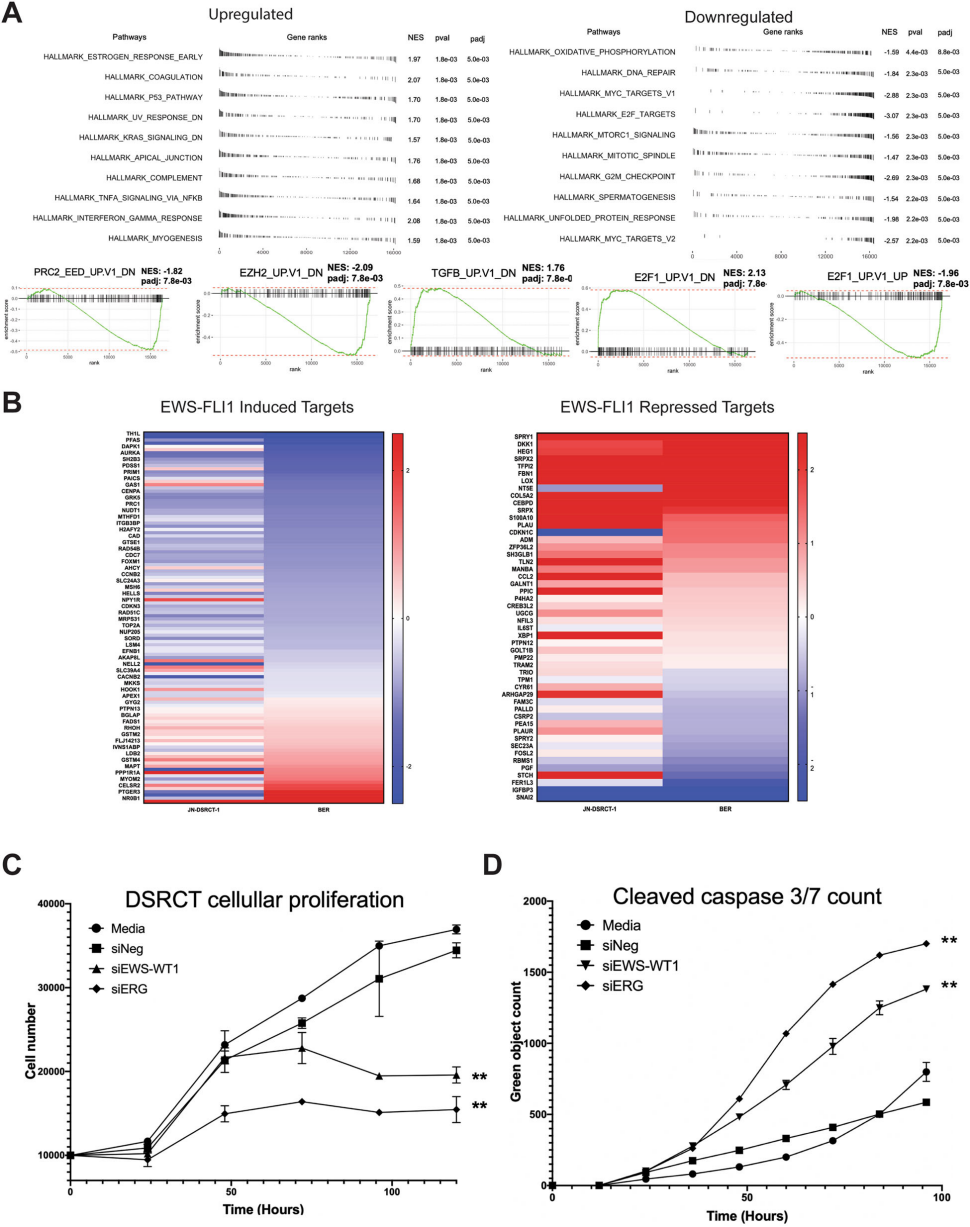
Identification of therapeutic vulnerabilities using functional gene set enrichment analysis (fgsea). **a** fgsea analysis of the top ten differentially regulated pathways in DSRCT cells following EWS-WT1 silencing demonstrates enrichment of cell-cycle and DNA repair pathways and highlights MYC signaling. There is also enrichment of gene sets that share similarities with the EWS-FLI1 transcription factor, including PRC2 complex-associated pathways, E2F, and TGFB. **b** Heatmaps showing either induction (red) or suppression (blue) of expression of known EWS-FLI1-induced (left) or repressed (right) targets with silencing of EWS-WT1 in DSRCT cells. Gene set enrichment analysis (fGSEA) was performed with functional gsea package (v1.10.1), and heatmaps were generated using pheatmap package (v1.0.12) in R (v3.6.1). **c** Silencing of ERG (siERG) leads to loss of cellular proliferation, which parallels that following EWS-WT1 silencing (siEWS-WT1) measured by the total cell number using MTS assay. Cell viability was measured using CellTiter96 (Promega) in three separate replicates plated in triplicate. **d** Silencing of ERG (siERG) leads to the accumulation of the apoptotic marker cleaved caspase similar to that seen following EWS-WT1 silencing (siEWS-WT1) measured using green fluorescent-cleaved caspase 3/7. siRNA and apoptosis measurement were completed as described above.

In order to better characterize the EWS-WT1 transcriptome, we used functional gene set enrichment analysis (fgsea) to evaluate the biologic pathways altered by EWS-WT1^11^. Importantly, we identified pathways that reflected known features of DSRCT. This tumor is known to be chemorefractive and is found primarily in males. Consistent with these observations, the fusion protein represses estrogen signaling, suggesting a dependence on androgen. In addition, fgsea identified strong pro-proliferative signatures, including the MYC signature identified in both cell lines (Fig. 4a). Finally, the tumor modulates the DNA damage response, both suppressing TP53 signaling and driving the expression of gene sets associated with the DNA damage response, suggesting a direct link between the fusion protein itself and resistance to chemotherapy.

Dysregulation of the DNA damage response is a prominent feature of other FET family fusions such as EWS-FLI1, the oncogenic driver of Ewing sarcoma. This correlation between EWS-WT1 and EWS-FLI1, despite different DNA-binding domains, suggests common mechanisms of tumorigenesis with these FET family fusions. Additional common features included an alteration in the E2F family of transcription factors, which has been previously described with EWS-FLI1, as well as modulation of other pathways dysregulated by EWS-FLI1, including TGFB and IGF signaling/mTOR^16–18^. It is notable that mTOR and IGF signaling have been previously identified as important in this disease^15,18^. An important observation is that many well-established EWS-FLI1 target genes were found to change in expression >0.5 log FC with EWS-WT1 silencing in DSRCT cells (Fig. 4b)^19^.

Finally, fgsea also suggested that EWS-FLI1 and EWS-WT1 might share common mechanisms of gene expression dysregulation. Most notably, we found a PRC2 signature that is known to be dysregulated by EWS-FLI1 in Ewing sarcoma cells^20^. In addition, EWS-WT1 upregulates the expression of ERG which is a close ETS family member of FLI1. It is possible that ERG is driving the expression of these targets, making this tumor an ETS-like tumor (Fig. 3c). Consistent with this idea, silencing of ERG leads to a striking loss of cell viability and induction of apoptosis that parallels the direct silencing of EWS-WT1 (Fig. 4c, d, Supplementary Figs. 2 and 3, Supplementary Table 5). Indeed, it appears that ERG is a critical component of the EWS-WT1 downstream transcriptional program, although more needs to be done to establish this idea.

In summary, we have used transcriptional profiling and functional gene set enrichment analysis to comprehensively characterize the transcriptome of EWS-WT1, thus providing the basis for ongoing investigations into the biology and therapeutic targeting of this tumor. We clearly demonstrate the dependence of DSRCT on the EWS-WT1 transcription factor for cell proliferation by selectively silencing EWS-WT1 in two primary DSRCT cell lines. We show reversal of proliferation and a late apoptotic cell death. In addition, we establish a comprehensive list of EWS-WT1 downstream targets, thus providing insight into the biology of the tumor, and highlighting important therapeutic vulnerabilities, including FGFR4, JAK3, mTOR, PDGF, ERG, and TGFB. This list of targets captures a number of therapeutic candidates that are both novel and previously identified by others using complementary approaches.

This study also shows that the fusion protein drives the expression of the DNA damage response as we identified a gene set called “Hallmark_DNA_repair” with a NES of −1.8 and a *P* = 0.0023. This gene set is known to have multiple members of both the nucleotide excision repair and transcription-coupled nucleotide excision repair pathways. In addition, EWS-WT1 directly represses TP53 signaling. These data may explain why this tumor is chemorefractory in the clinic. More importantly, it suggests that a therapeutic vulnerability may exist in one of the DNA damage response pathways in this tumor, either at baseline or with suppression of EWS-WT1.

Interestingly, this study also makes the novel observation that there is common biology shared with other fusion proteins, and EWS-FLI1 in particular, despite structural differences in the DNA-binding domain of the two proteins. While the mechanism is not clear, EWS-WT1 upregulates the expression of ERG which is a close family member to FLI1. This may be the reason why so many common targets were identified. Importantly, the cells show a striking dependence on ERG expression, suggesting that these gene expression changes are fundamentally important in the dependence of DSRCT on EWS-WT1. However, there may also be common epigenetic mechanisms that have been well-described for EWS-FLI1, such as the dysregulation of PRC2 as was suggested by the fgsea analysis.

Most importantly, this study definitively shows a dependence of these cells on EWS-WT1 for continued proliferation in two different models to establish the fusion protein as a therapeutic target for DSRCT.

## Supplementary information

Supplemental Figure 1

Supplemental Figure 2

Supplemental Figure 3

Supplemental Table 1

Supplemental Table 2

Supplemental Table 3

Supplemental Table 4

Supplemental Table 5

## Data Availability

The sequencing data have been deposited on GEO under the accession number GSE137561.
