# Supplementary figures and images for: Desmoplastic small round cell tumor is dependent on the EWS-WT1 transcription factor
Source: Oncogenesis. 2020 Apr 28;9(4):41. doi: 10.1038/s41389-020-0224-1 (PMC7188842; doi:10.1038/s41389-020-0224-1)

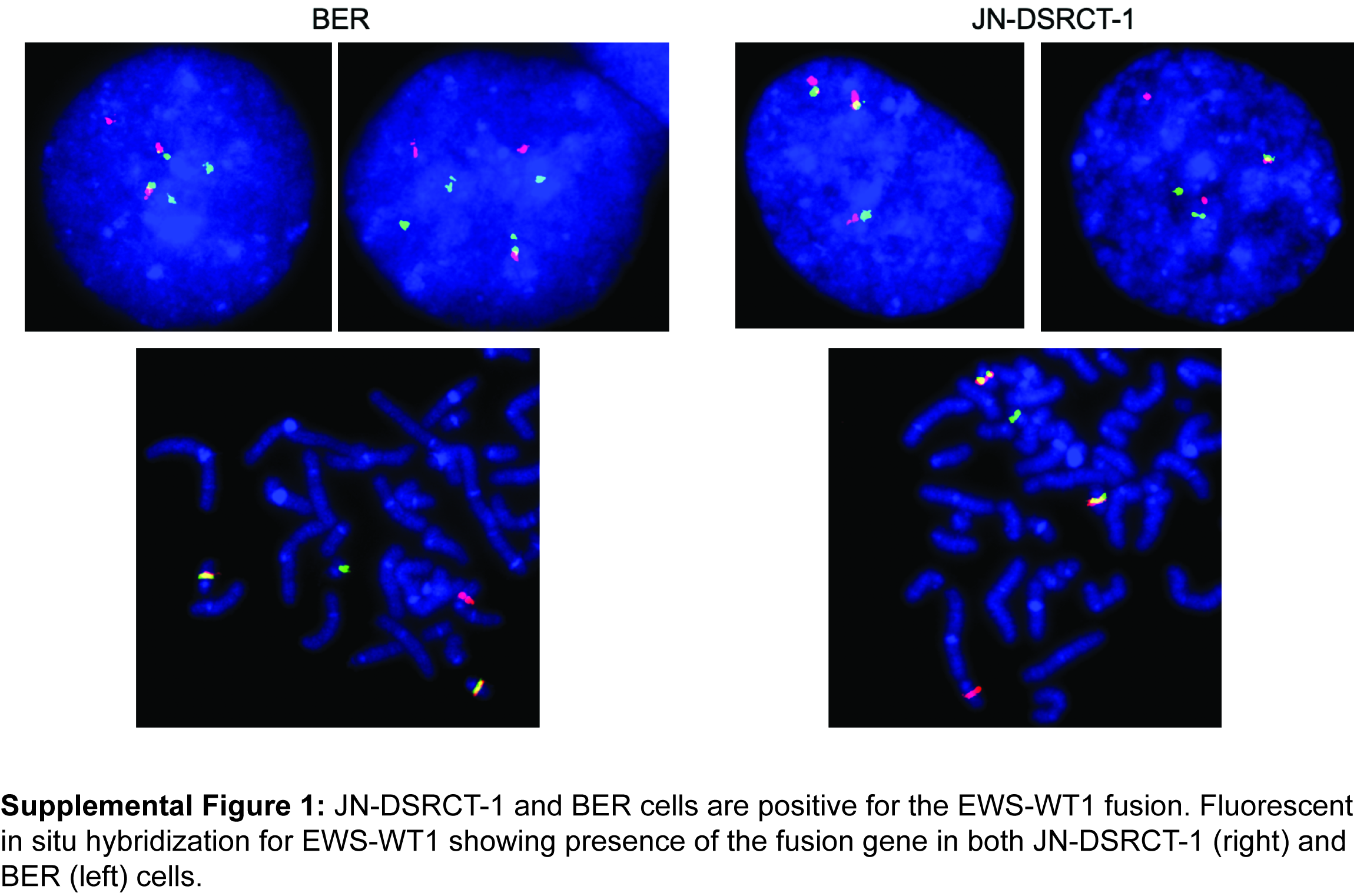

Supplement: Supplementary file 1 — Supplemental Figure 1 [file 41389_2020_224_MOESM1_ESM.tif]

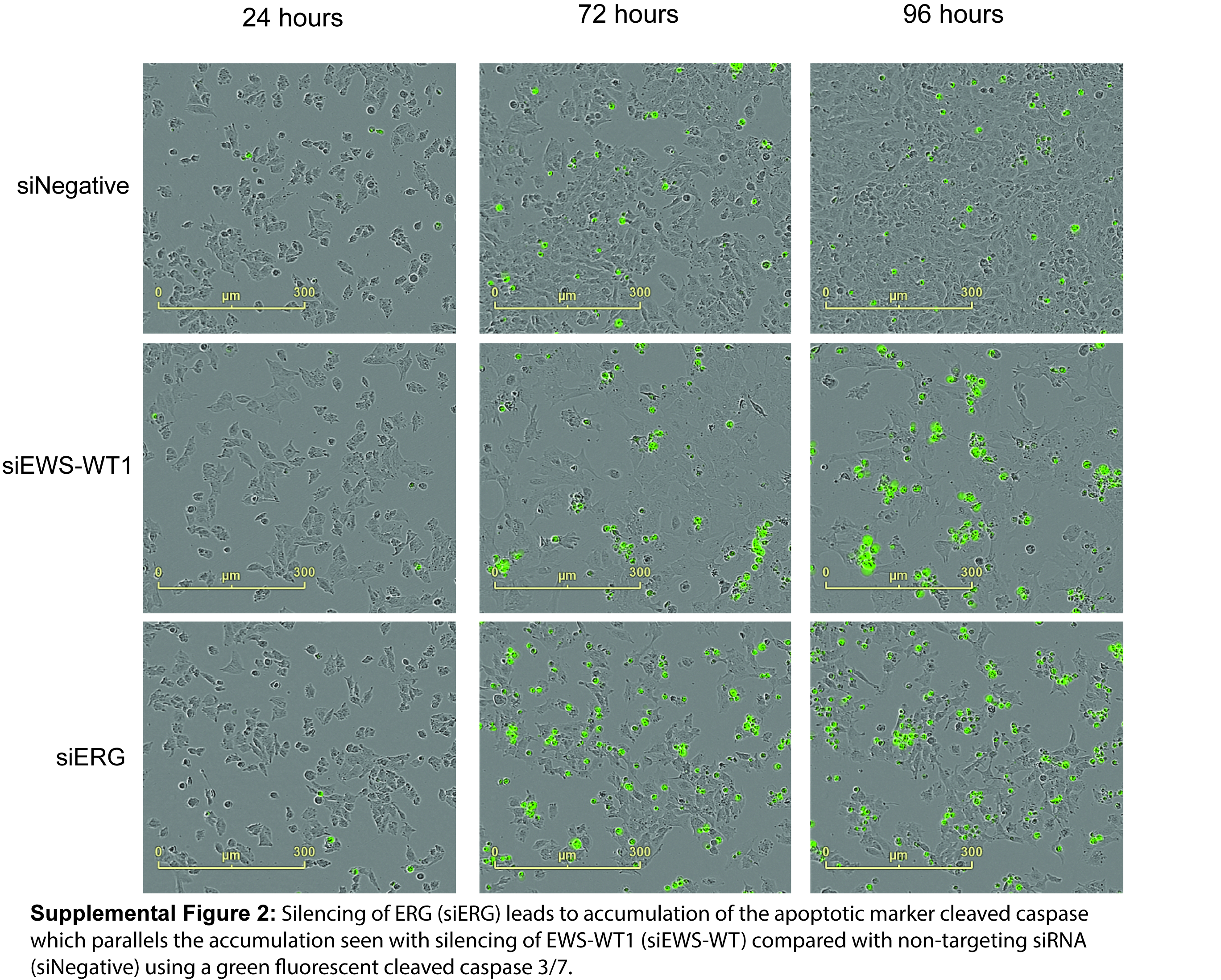

Supplement: Supplementary file 2 — Supplemental Figure 2 [file 41389_2020_224_MOESM2_ESM.tif]

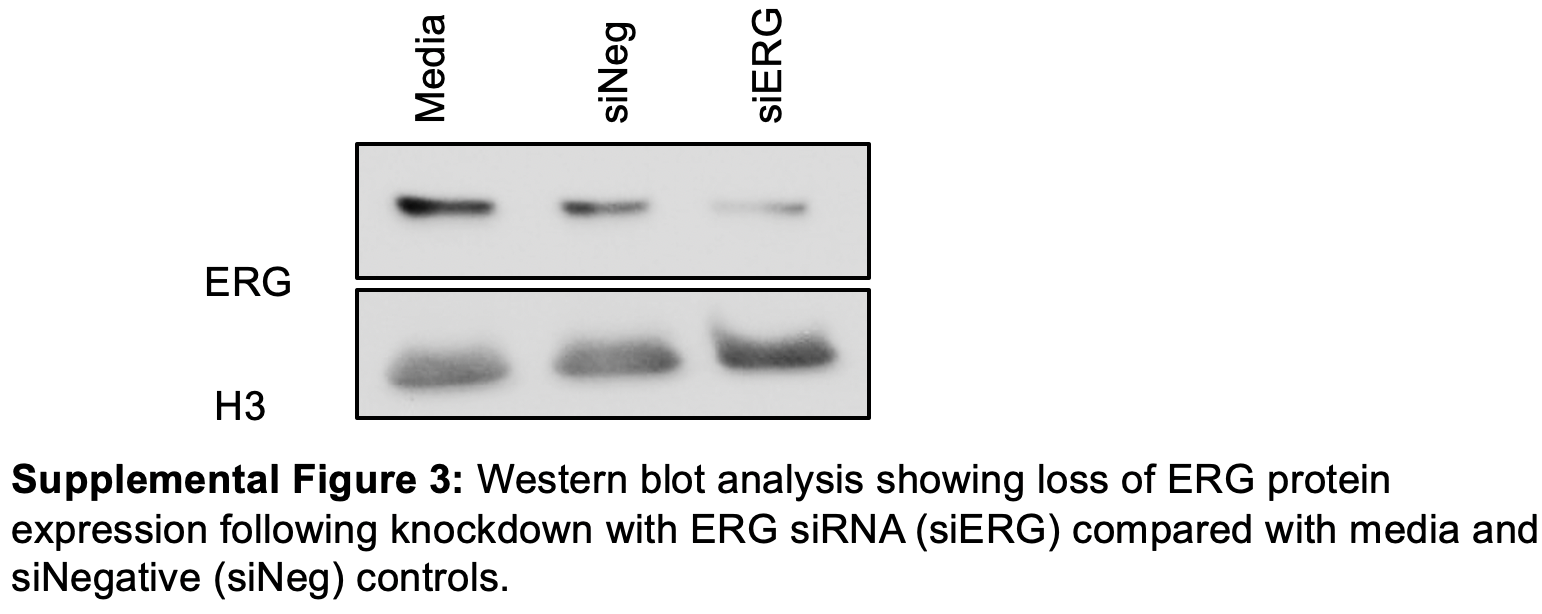

Supplement: Supplementary file 3 — Supplemental Figure 3 [file 41389_2020_224_MOESM3_ESM.tif]
